# MeshID: Few-Shot Finger Gesture Based User Identification Using Orthogonal Signal Interference

**DOI:** 10.3390/s24061978

**Published:** 2024-03-20

**Authors:** Weiling Zheng, Yu Zhang, Landu Jiang, Dian Zhang, Tao Gu

**Affiliations:** 1 School of Computing Technologies, RMIT University, 124 La Trobe Street, Melbourne, VIC 3000, Australia; zwlcle@hotmail.com; 2 School of Computing, Macquarie University, 4 Research Park Drive, North Ryde, NSW 2109, Australia; y.zhang@mq.edu.au; 3 Base of Red Bird MPhil, HKUST(GZ) University, No.1 Du Xue Rd., Guangzhou 511458, China; landu.jiang@mail.mcgill.ca; 4 College of Computer Science and Software Engineering, Shenzhen University, 3688 Nanhai Blvd, Shenzhen 518060, China; zhangd@szu.edu.cn

**Keywords:** device-free behavioral sensing, orthogonal signal interference, user identification

## Abstract

Radio frequency (RF) technology has been applied to enable advanced behavioral sensing in human-computer interaction. Due to its device-free sensing capability and wide availability on Internet of Things devices. Enabling finger gesture-based identification with high accuracy can be challenging due to low RF signal resolution and user heterogeneity. In this paper, we propose MeshID, a novel RF-based user identification scheme that enables identification through finger gestures with high accuracy. MeshID significantly improves the sensing sensitivity on RF signal interference, and hence is able to extract subtle individual biometrics through velocity distribution profiling (VDP) features from less-distinct finger motions such as drawing digits in the air. We design an efficient few-shot model retraining framework based on first component reverse module, achieving high model robustness and performance in a complex environment. We conduct comprehensive real-world experiments and the results show that MeshID achieves a user identification accuracy of 95.17% on average in three indoor environments. The results indicate that MeshID outperforms the state-of-the-art in identification performance with less cost.

## 1. Introduction

Human Activity Recognition (HAR) and Human Behavior Recognition (HBR) technologies are integral components of Human-Computer Interaction (HCI) systems. They enable computers to interpret and respond to human actions and behaviors, enhancing the overall user experience. HAR and HBR systems [1] utilize various sensors and algorithms to analyze data such as movement patterns, gestures and physiological signals, facilitating seamless interaction between humans and computers. However, they raise security concerns about potential misuse or unauthorized access to users’ data. Through robust user identification methods such as biometrics, passwords, or behavioral analysis, HCI systems can mitigate the risk of unauthorized access. Vision technology [2,3] can identify different users through physical activity characteristics captured from image frames using high resolution cameras, but it is susceptible to failure in the presence of luminous changes and obstacles within the line-of-sight (LoS) [4], thus exacerbating significant concerns regarding user privacy. In stark contrast, RF sensors (such as WiFi, RFID, and Zigbee) offer numerous advantages, including freedom from illumination constraints, reduced privacy apprehensions, equitable penetration and widespread availability on IoT devices. As a result, they are widely proposed to enable advanced device-free behavioral sensing [5,6]. Towards RF-based behavioral sensing, existing systems propose a variety of behavioral characteristics including daily activities [7,8,9,10], vital signs [11,12] and gestures [13,14,15]. Although these systems have demonstrated their effectiveness with fair accuracy in laboratory settings, they may still encounter real-world constraints. Extracting biometric features from daily activities usually require users consistently performing a set of pre-defined activities for long-term tracking [5]. The motion of vital signs (e.g., heartbeats [16] and respiration rate [17]) remains fragile, and identification may be prone to failure due to body movement artifacts and ambient noise. In contrast, performing gestures (e.g., finger gesture) for identification can practically mitigate the impact of motion noise and offer considerable user-friendliness [18,19,20]. Gesture-based interaction stands as the most common and efficient method of HCI [21,22,23]. Gesture recognition technology is mature and capable of achieving high accuracy. When users write in the air, they commonly use their forefinger naturally. In our experiment, we adhere to this habit by employing forefinger gestures for user identification. Enabling user identification through finger gestures with high accuracy is a non-trivial challenge, which requires addressing two key challenges.

Firstly, as the movement of the finger motion is quite small, the amplitude change in the RF reflected signal caused by finger motion could be very faint, hence it is difficult to identify minor variance among users’ biometrics extracted from the limited signal variance. According to the theory of the Fresnel model [24,25], when a user moves his/her hand across the boundaries of Fresnel zones, the CSI of the signal will form a series of peaks and valleys. We regard this variance pattern as a kind of RF signal resolution. If we have more peak values in an unit area we can say that the RF signal resolution is higher. Once the motion becomes smaller, like finger motion, the number of peak values is reduced and the amplitude change in CSI diminishes much smaller, resulting in low accuracy for identification. The key question we have is how to fundamentally improve the RF signal resolution (i.e., CSI variance). Inspired by Young’s double-slit interference experiment [26], we use a pair of transmitters (double-source) in the same frequency to induce the signal interference. The double-source interference produces numerous dense narrow beams. In other word, it greatly increases the number of boundaries in the same unit area compared with traditional methods. By setting up two orthogonal double-source pairs, the sensing area will be covered by a dense signal mesh, hence the signal resolution is enormously improved.

Secondly, due to user heterogeneity (e.g., different users, preferences, and surroundings), in reality, the data distribution of users’ biometrics may become complex and un-predictable to fail user identification. The performance of the traditional deep learning(DL) technique relies heavily on collecting a large amount of user data as a prerequisite, especially assuming that the represented data distribution is relatively stationary without dynamic changes. Towards robust and efficient model retraining, we utilize a one-shot learning approach based on the Siamese network [27,28,29], with two core techniques: first component reverse (FCR) extraction and convolution block attention module (CBAM), achieving high model robustness and performance in heterogeneous scenarios (e.g., identifying unseen users). A unique velocity distribution profiling (VDP) is calculated from a double-source interference pattern, reflecting the personal motion features.

When users perform finger gestures in a complex environment, the input feature space of extracted biometrics contains both non-related features (i.e., common features shared by the same gestures of users and ambient noise) and user-specific features (i.e., personal features), but the issue is that the non-related features may strongly affect the performance of identifying users. To improve it, we design a first component reverse (FCR) extraction, inspired by principal component analysis (FCR) extraction, inspired by principal component analysis (PCA), hat removes the non-related features (i.e., first component in PCA) and helps extract user-specific features from the input feature space, boosting our CBAM-based Siamese network with a superior identification capability.

To address the above issues, we propose MeshID, a novel RF-based user identification approach leveraging signal interference for accurate finger gesture tracking. MeshID is able to significantly improve the sensing sensitivity by leveraging double-source signal interference and extracting subtle individual biometrics from less distinct finger motions. Due to the effect of enhancing CSI variance, our mesh approach can mitigate the multipath effects and contribute to resisting the interference from ambient environments. By applying an efficient CBAM-based few-shot deep learning framework with FCR extraction, MeshID achieves high model robustness and can be easily adapted to new users with complex surroundings.

The main contribution are summarized as follows:

- We present MeshID, an RF-based user identification approach based on a beam structure caused by double-source orthogonal signal interference. The system is able to detect user in-air finger motion with high accuracy, which could support for a variety of smart home/building applications especially for user identification.
- To the best of our knowledge, MeshID is the first solution that derives the unique velocity distribution profiling (VDP) features from signal patterns leveraging the novel mesh beam structure. It fully enables user identification by essentially enhancing the signal sensing sensitivity compared to traditional RF-based approaches
- We design a first component reverse (FCR) extraction method to emphasize user-specific features and remove non-related features, hence improving the identification accuracy and model capability. We propose a one-shot learning framework with CBAM in a Siamese network for model retraining robustness and efficiency.
- We evaluate MeshID in comprehensive real-world experiments. The results demonstrate that MeshID is able to achieve an average identification accuracy of 95.17% on average by performing single finger gestures in three indoor environments.

The rest of the paper is organized as follows. Section 2 discusses the preliminary knowledge of the method. Section 3 presents the design of MeshID. Section 4 demonstrates the comprehensive evaluation results. Section 5 shows the key discussions. Section 6 reviews the related works. Section 7 concludes the paper and discusses future works.

## 2. Preliminary

In this section, we introduce the fundamental concept of Channel State Information (CSI) and then explore the double-source interference phenomenon for finger gesture-based user identification.

CSI is a fine-grained physical layer that depicts how RF signals propagate between a transmitter and a receiver [30,31]. It captures the slight change in the surrounding objects in both the time domain and spatial domains. The CSI channel *H* is modeled by Y(f,t)=H(f,t)X(f,t)+N, where Y is the received signal, X is the pre-defined modulated signal and N is the noise vector.

**Figure 1:**
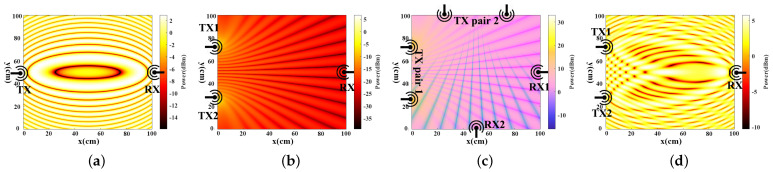
The simulation heatmaps: (**a**) Fresnel zones from single source; (**b**) Interference patterns from double sources; (**c**) Mesh of the formed by orthogonal signal interference; (**d**) Fusion of interference and Fresnel zone from double sources.

Empirical Study of double-source interference. In tradition, for a pair of transmitters and receivers, as shown in Figure 1a, the signal variance pattern caused by the reflection of target activity is usually identified as Fresnel zones. Small finger motions in the same zone usually have a small impact on the signal variance, while user activity across different zones causes a large signal variance. We may regard these zones as a kind of “sensing sensitivity”. The zone number can be a measure of sensitivity to sensing. For example, in a 1×1 m area (without losing generality, the frequency is set as 5.76 GHz), roughly we may have only seven Fresnel zones.

To increase the boundaries of sensing, our intuition is to apply RF signal interference. Inspired by Young’s double-slit interference experiment [26], we use a pair of transmitters with same frequency to induce the signal interference, resulting in a stripe pattern in a parallel way. The simulation results of signal reflection are depicted in Figure 1b. The reason why we can see such a fringes pattern is that multiple sources signals can interfere with each other constructively, where the amplitude of combined signal is greater than the individual one, while interference destructively where the amplitude of the combined signal is smaller than the individual one or same with the original. We call the above phenomenon double-source interference. Double-source interference obeys the Huygens-Fresnel Principle [32], hence the Fresnel zones still exist under this double-source setting, as shown in Figure 1d. The double-source fringes pattern and the Fresnel zones are overlapping with each other, the Fresnel zones will be divided into several “mesh” cells. Within this dense mesh, if use activity is crosses the cell borders, it also causes a larger signal variance. That is, in the same 1×1 m area, we may roughly have more than 83 separated areas in total. The boundaries of “mesh” with fringes are notably increased by interference compared to the Fresnel zones, which indicates that the sensing sensitivity on RF signal interference increases. The interference of multiple waves are generally regarded as a not-so-trivial negative effect leading to unpredictable signal patterns and poor signal quality in wireless sensing, many studies aim to mitigate interference for better accuracy. However, our objective is to utilize such negative effect in user identification and change the effect from “negative” to “positive”.

User identification based on orthogonal double-source interference: Since that a finger gesture may lead to motions in different directions (e.g., drawing a digit “0”), one transmitter pair may only be sensitive to the motions in the perpendicular direction to the transmitters, hence it may lose useful motion features. To address this, we propose a new setup of orthogonal antenna pairs to enable a dense interference pattern (i.e., dense mesh) from both vertical and horizontal directions. Our basic idea is that if we deploy another pair of transmitters with the same distance, but in another direction (e.g., parallel to the *x*-axis) and with a slight frequency difference, such transmitters also cause a stripe result but in a vertical way. The amplitude of the received signal appears like a dense mesh with two orthogonal interference transmitter pairs, as shown in Figure 1c. We name such pairs of transmitters as orthogonal antenna pairs. To observe the mesh pattern of orthogonal double-source interference clearly, the Fresnel zone is omitted from this figure. Consequently, we are able to achieve a higher sensing sensitivity on RF signal compared to traditional methods.

**Figure 2:**
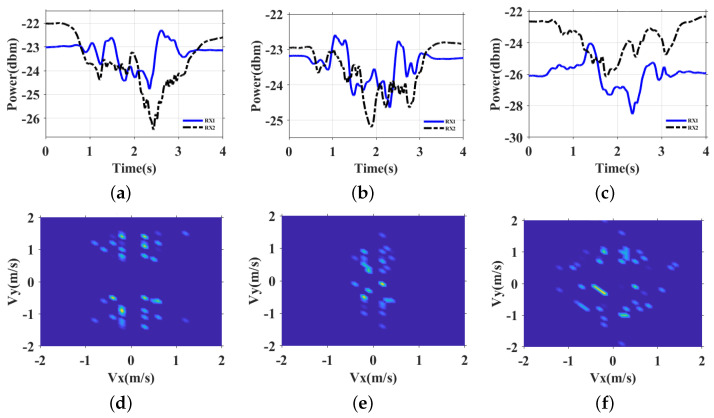
The VDP of different users when writing digit 0. (**a**) CSI of user 1. (**b**) CSI of user 2. (**c**) CSI of user 3. (**d**) VDP of user 1. (**e**) VDP of user 2. (**f**) VDP of user 3.

The received CSI comprises a mixture of signals, including the line-of-sight component, double-source component, and others. We utilize the Complementary Ensemble Empirical Mode Decomposition (CEEMD) to separate the double-source interference component from the CSI data. With this feature, we propose a noval VDP feature to capture the fine-grained finger motions of users within the sensing area. The VDP encapsulates the motion change pattern of the user and the corresponding potential biometric features such as the motion speed. Since users have diversity motion habits and behavioral habits (e.g., drawing digit “0” clockwise or anticlockwise, different pauses and speeds when performing gesture), even when executing the same gesture, the moving finger interacts with RF signals differently, resulting in distinct patterns. As shown in Figure 2, Figure 2a–c are the CSI variances of three different users drawing the same digit “0” based on their own writing habits. Figure 2d–f are the corresponding VDPs that have different velocity patterns. Therefore, we can depict users’ behavioral characteristics by leveraging biometric feature extraction methods and bring the opportunity for user authentication.

## 3. Methodology

### 3.1. Framework

**Figure 3:**
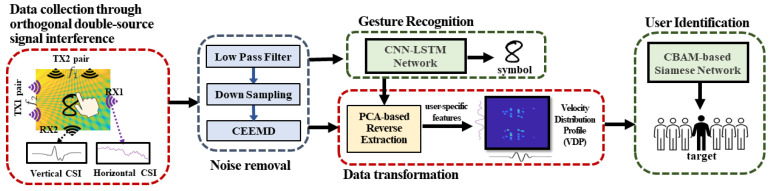
MeshID design overview.

As shown in Figure 3, our design consists of four components: (1) data collection from orthogonal double-source interference, (2) noise removal and gesture recognition, (3) FCR extraction analysis to extract user-specified features, (4) data transform and (5) CBAM-based few-shot learning for identification.

Firstly, we introduce our design of the orthogonal signal interference. The signal interference pattern is like a dense mesh. The user is able to perform finger gestures within this area. Under this setting, CSI information is collected from both horizontal and vertical transmitters. Secondly, a low pass filter algorithm is used to remove the high-frequency noise and a down sampling algorithm is used to reduce the data size in order to effectively process the data. Then, we use Complementary Ensemble Empirical Mode Decomposition (CEEMD) [33] on the data to obtain ensemble Intrinsic Mode Function (IMF) which is related to the interference pattern. Thirdly, with the IMF data from the CSI series, we utilize a CNN-based LSTM method to recognize the user’s gesture. Then, we leverage FCR extraction to remove the effect of the shared component, so that it can remove the correlated non-related features among different users and leave the user-specified features of the user. Fourthly, we estimate the user instantaneous moving speed according to the fringe-based variance pattern, then we generate the VDP for following user identification. Finally, we can identify users by leveraging the CNN-based Siamese Network.

### 3.2. Double-Source Interference

In free-space scenarios, where two RF waves with the same frequency *f* travel along two different paths to converge at the same destination point, the difference of two path lengths is equivalent to a multiple of wavelength λ, which is known as constructive interference [34] Similarly, if the two waves are out of phase by half of the wavelength, the result is destructive interference. In this case, the combined signals produce several fringes, where the high points of fringes are called the crests, and the low points of the fringes are called the troughs.

Given this phenomenon, we may deliberately utilize the aforementioned double-source signals, each possessing the same radio frequency, to create a combined signal $H_{sum}$, which can be defined as follow:

(1) $$H_{sum}\left(f,t\right)=H_{1}\left(f,t,r_{1}\right)+H_{2}\left(f,t,r_{2}\right)$$

where $H_{1}$ is the received signal travel with distance $r_{1}$ from the first antenna, and $H_{2}$ is from the second antenna and travels with distance $r_{2}$.

**Figure 4:**
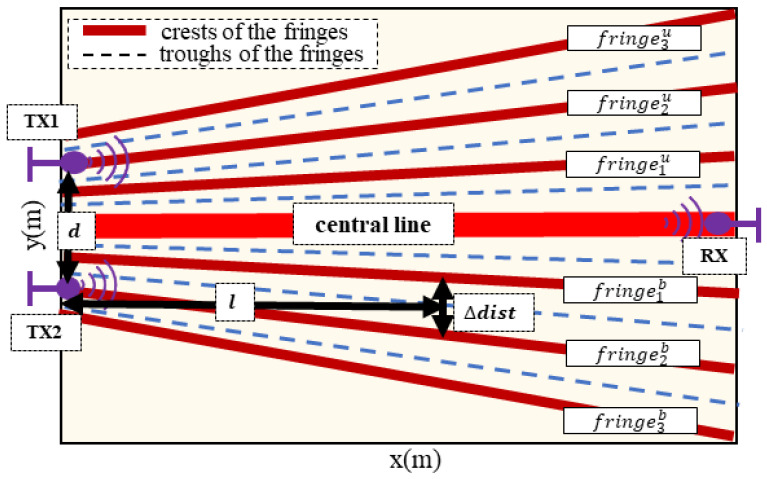
Illustration of double-source interference.

The width of the neighbouring fringes can be calculated by three primary major parameters: the distance between the transmitter node pairs, the wavelength of the radio wave, and the distance from the transmitter pairs. The node distance between nodes and the wavelength of the radio wave are the known values. The impact range of finger motion usually is small compared to the sensing area, hence the distance from the transmitter pair can be regarded as the middle of the sensing area. The fringes are symmetrical starting from the central line (thick red line in the figure) of the receiver antenna, we refer to the upper fringes as $fringe_{m}^{u}$ and bottom fringes as $fringe_{m}^{b}$, *m* is the number of a specific fringe number, as shown in Figure 4. The width Δdist between fringe $fringe_{m}^{u}$ and $fringe_{m+1}^{u}$ (or between fringe $fringe_{m}^{b}$ and $fringe_{m+1}^{b})$ at a position whose distance to the *y* axis is *l*, can be calculated by

(2) $$\Delta dist=m\lambda \sqrt{4+\frac{l^{2}}{d^{2}-m^{2}\lambda^{2}}}$$

where *d* is the node distance between the transmitter antennas pairs, λ is the wavelength.

### 3.3. Orthogonal Signal Interference

In previous instances of interference fringes, employing a pair of antennas in transmission resulted in fringes appearing predominantly along one direction (e.g., horizontal direction in Figure 1b). Introducing another pair of antennas orthogonally (e.g., vertically, as shown in Figure 1c), operating at slightly different frequencies, produces interference fringes along a different axis (vertical direction). Therefore, the combined interference signal fringes appear like a mesh. We can effectively capture the fine-grained finger motion from any direction, thereby enhancing signal resolution.

We may have CSI data from both vertical and horizontal antenna pairs at the same time. The orthogonal RF signals can be represented as below:

(3) $$M\left(RX_{1},RX_{2}\right)=\left[\begin{array}{c} H_{RX_{1}}\left(f,t\right)\\ H_{RX_{2}}\left(f,t\right) \end{array}\right]$$

where $H_{RX_{1}}$ is the received signal at receiver RX1, and $H_{RX_{2}}$ is the received signal at receiver RX2.

### 3.4. Noise Removal and Gesture Recognition

The raw wireless signals are inherently noisy due to the multipath effect. In order to effectively identify the human, we should remove noise first, then perform the data transformation for enhanced analysis.

#### 3.4.1. Low Pass Filtering

The CSI variance caused by human motion has a relatively low frequency specified as $f_{l}$, while the high-frequency data usually contain environment noise. Therefore, we utilize a a low-pass FIR filter to remove the environmental noise. The cut-off angular frequency used in the low pass filter is calculated as $\omega_{c}=2\pi \frac{f_{l}}{\tilde{f_{s}}}$.

#### 3.4.2. Down Sampling

To expedite calculations and enhance processing speed, we initially employ a down-sampling (interpolation) method to stretch the input CSI matrix. Assuming the original sampling rate is $f_{s}$, and the data length of the original data is *m*. After the re-sampling, the data length is *n* with the new sampling rate $\tilde{f_{s}}=f_{s}*\frac{m}{n}$. The CSI matrix after re-sampling can be rewritten as $\tilde{M}\left({\tilde{f}}_{s}\right)=F\left(M,f_{s}\right)$ where M is the input orthogonal RF signal.

#### 3.4.3. Interference Pattern Extraction

**Figure 5:**
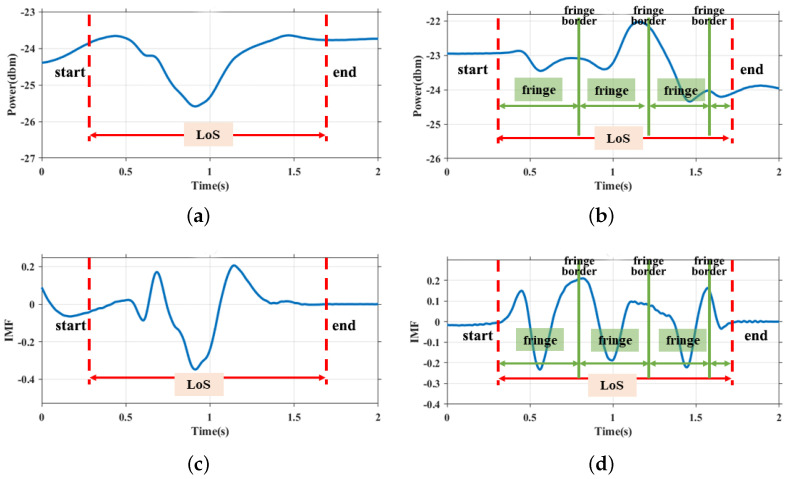
Comparison of Fresnel Zones patterns and interference patterns when user’s finger moves perpendicular to the LoS. (**a**) CSI of 1st Fresnel zone with single source. (**b**) CSI of Interference fringes with double sources. (**c**) IMF of 1st Fresnel zones with single source. (**d**) IMF of Interference fringes with double sources.

Given that interference theory remains constrained by the principles of the Fresnel Zone, the interference pattern can be covered by the Huygens-Fresnel Principle. Figure 5 shows an example of how both the Fresnel zone and environment noise can detrimentally impact the interference pattern. We place the receiver antenna on the right side of the drawing area. The transmitter is placed on the left side. Horizontal zones/fringes will exist with a single sourcesetting/double source setting, as shown in Figure 1a,b. While a user draws a straight line from center top to center bottom and just crosses the Line-of-Sight (LoS), the amplitude variance with a single source setup is shown in Figure 5a, we identify the start point and the endpoints with a red dashed line. The figure clearly shows that only one peak value is shown within this range. For purposes of a fair comparison, we repeat the same finger motion under a double-source interference setup. We also identify the start point and end point with red dashed lines. The amplitude variance is shown in Figure 5b. The result indicates that three peak values are distributed in the same range compared with a single source. We utilize a green line to separate the interference fringes based on the peaks. On the basis of Equation (2), we could estimate the width of the fringes. Theoretically, motion features based on personal velocity can be derived if the boundaries of the fringes are accurately identified. Therefore, there is a pressing need for an efficient method to detect these fringe boundaries with precision.

We apply Complementary Ensemble Empirical Mode Decomposition (CEEMD) on the CSI matrix $\tilde{M}$ to detect the fringe boundaries.

(4) $$\tilde{M}\left(t\right)=\sum_{i=1}^{N}f\left({IMF}_{i}\left(t\right),\varepsilon_{i}\right)+res_{N}\left(t\right)$$

where $f({IMF}_{i})$ is the ith Intrinsic Mode Function (IMF ) component, $\varepsilon_{i}$ is the residue of added white noises, and $res_{N}\left(t\right)$ is the residue.

To simplify the pattern extraction, we can roughly divide the received CSI signals into four layers: noises, interference variances, Fresnel zone variances and trends related to with the distance of transmitter and target objects. Hence, N=3 in our system, and the residue of the decomposition represented the trend of the CSI series. We will use ${IMF}_{2}$ as the input of the following calculation. The results of CEEMD processing are shown in Figure 5c and Figure 5d respectively.

### 3.5. Finger Gesture Recognition

In this subsection, we describe the gesture recognition methods to recognize users’ finger gestures. Unlike some existing work, they require the collection of lots of gesture samples for training the gesture recognition network to meet the requirement for real-world applications that may have few gesture samples for first-time usage. Also, the recognition system is required to offer a short response for real-time applications. We employ Convolutional Neural Network (CNN) based Long Short-term Memory (LSTM) for finger gesture recognition. In our system, only a few gesture samples of a user are needed for re-training the network. The gesture recognition network contains four 1d CNN layers to suppress the data size to shorten the training time. Next, the features are fed into a two-layer LSTM. Based on the known gesture, we use a general Principal Component Analysis (PCA) algorithm to analyze the CSI value for removing the common features of the same gesture.

### 3.6. First Component Reverse Extraction

**Figure 6:**
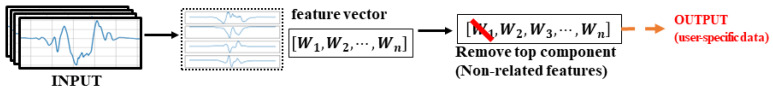
Architecture of FCR extraction.

Principal Component Analysis (PCA) has been widely used for signal denoising to enhance classification performance [35,36]. Most of the existing works usually keep the top three principal components (especially the first component) of the data and ignore the rest, as PCA ranks the principal components in descending order in terms of their variance.The design of a first component reverse (FCR) extraction method is shown in Figure 6.

**Figure 7:**
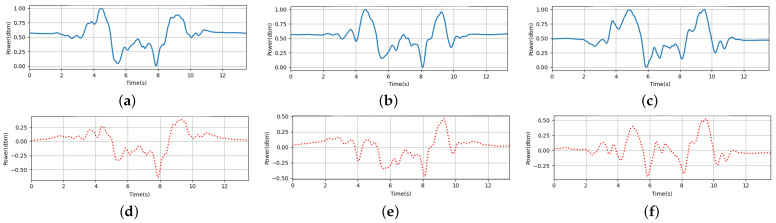
When three different users perform same gesture (digit “8”), the comparison of CSI amplitude before and after FCR extraction. (**a**) User 1 (before FCR extraction). (**b**) User 2 (before FCR extraction). (**c**) User 3 (before FCR extraction). (**d**) User 1 (after FCR extraction). (**e**) User 2 (after FCR extraction). (**f**) User 3 (after FCR extraction).

In our user identification scenario, the first component derived from the received CSI data of the same finger motion may majorly contain the common features shared among different users when they are performing the same gestures. As shown in Figure 7a–c, when three users perform the same gesture digit “8”, the major CSI variance from the same gesture is quite similar. The major CSI variance is caused by the finger motion. We recognize the first component of PCA from these similar variances as the common feature. It is a non-related feature for user identification. Instead, our aim is to extract user-specified features. After remove the non-related features, the rest variances are influenced by diverse body structures, environmental factors, and other sources of noise. Although non-related features may aid in recognize the defined finger gestures across different people, they can adversely impact user identification. We aspire to incorporate more user-specific features for accurate identification. As shown in Figure 7, the CSI result of three different users are Figure 7a, Figure 7b and Figure 7c, respectively. All three users are writing the digit “8” freely. Figure 7d–f are the corresponding CSI by applying FCR extraction. The first component of different users is almost similar to each other. Upon removing these similar components, user-specific features become more pronounced. Therefore, in our design, we remove the principal component to effectively reduce the impact of such non-related features, and extract the characteristic of personal information (i.e., user-specified features) accordingly. This methodology aids in identifying different users by filtering out interference from non-related features without sacrificing personalized information.

Applying the FCR extraction algorithm, we will have a coefficient matrix $\left[W_{1},W_{2},\ldots ,W_{n}\right]$. We remove the first component to enhance the performance of one-shot learning for user identification

(5) $$X=X_{IMF}-X_{IMF}\times W_{1}$$

where $W_{1}$ is the coefficient of first component and $X_{IMF}$ is the IMF component of CSI.

### 3.7. Interference-Based Velocity Distribution Profiling

In observational experiments, we notice that users exhibit brief pause intervals during gesture execution, causing fluctuations in instance motion velocity. Each user possesses a unique instance velocity profile due to his/her inherent writing behavior. We calculate the user velocity profile based on the $x_{IMF_{n}}$ by analyzing the interference pattern. It’s been observed that when a target user moves his/her finger across from the boundary of an interference fringe to the middle part of the neighbour interference fringe, a maximum value $A_{i}^{max}$ and a minimum value $A_{i}^{min}$ of the CSI amplitude occur in corresponding CSI time series, as shown in Figure 5d. For two neighboring extreme values, we derive the width of the fringe Δdist which is calculated by Equation (2). Hence, the instantaneous velocity is

(6) $$v_{\left(inst\right)}\left(t\right)=\frac{\Delta dist}{\Delta t}$$

where Δt is decided by the sampling rate.

For each receiver, we segment the signal with a small time window $win_{l}$, and calculate the velocity profile by the interference fringes. The two velocity profiles from two receivers RX1 and RX2 can be identified as the horizontal velocity profile $V(v_{x})$ and vertical velocity profile $V(v_{y})$, because in the MeshID system, receivers are perpendicular to each other. For each time window, we search for the extreme values of the one-dimensional IMF time series to identify the corresponding fringe boundaries, and use them to derive the instantaneous velocity $V(v_{x},v_{y})$. A two-dimensional VDP matrix VDP[M×N] with size [M×N] by quantizing the discrete instantaneous velocity from horizontal and vertical directions. The VDP combines the velocity distribution of the two velocity profiles. VDP can effectively extracts the real-world features of the users since it reflects real-world movements. On one hand, when a user performs the same gestures at different movements with fixed antennas but different movements, the corresponding VDPs are similar to each other. On the other hand, as Figure 2 shows, VDP is different from user to user.

### 3.8. CBAM-Based Siamese Network

**Figure 8:**
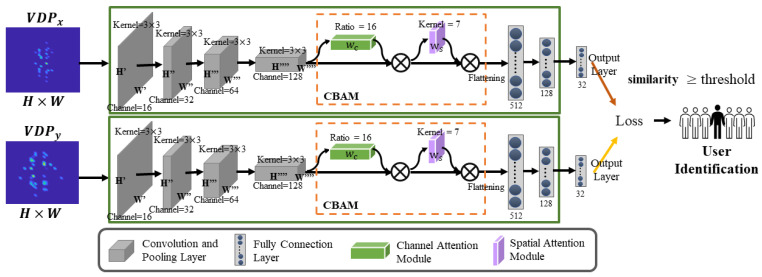
Few-shot learning overview.

In order to facilitate efficient model retraining for addressing user heterogeneity, we leverage the Siamese neural network for few-shot learning in user identification. It aims to train only few data from unseen users, requiring less model retraining preparation to achieve satisfactory performance. Additionally, the few-shot learning takes advantage from features of previously learned VDP samples, showcasing its capability to identify new individuals with reduced effort. As shown in Figure 8, our proposed model comprises two twin networks that share same parameters and weights.

The purpose of the Siamese network is to minimize the pairwise distances between personal drawing features from the same people and maximize the distances of features from different people. The process can be illustrated as follows:

(7) $$\delta \left(X_{\left(i\right)},X_{\left(j\right)}\right)=\;\left\{\begin{array}{cc} \min\;∥F\left(X_{\left(i\right)}\right)\;-\;F\left(X_{\left(j\right)}\right)∥,\;\;\; & \;U_{\left(i\right)}\;=\;U_{\left(j\right)}\\ \max\;∥F\left(X_{\left(i\right)}\right)\;-\;F\left(X_{\left(j\right)}\right)∥,\;\;\; & \;U_{\left(i\right)}\;\neq \;U_{\left(j\right)} \end{array}\right.$$

where F is a non-linear transform based on a twin network.

Particularly, we adopt the Convolutional Block Attention Module (CBAM) [37] in our Siamese network to emphasize extracting informative features along both the channel and spatial axis. CBAM is an effective attention module for most feed-forward CNN networks. For a given feature map $\Upsilon_{s1}$, CBAM calculates its channel and spatial weight matrix sequentially and then refines the feature map based on these two weight matrices. Specifically, the Siamese network uses the two same networks to extract features, examine the similarity of the input anchor VDP image and store the VDP image for user identification. Therefore, though the CBAM-based Siamese network with a learned VDP feature of an individual, MeshID can authenticate users with a high identification accuracy.

In the training phase, the VDP matrix/spectrogram with size [M×N] triplets (anchor VDP image, negative VDP image, positive VDP image) are fed into the CBAM-based Siamese model. The basic idea is that the distance between drawing patterns of the same person on the same character should be smaller than that between the drawing patterns of different people. We take combined VDP (i.e., horizontal antenna pair RX1 and vertical antenna pair RX2) as inputs to each stream (sub-network). The architecture of each sub-network is mainly divided into three modules.

More specifically, the input VDP matrix/spectrogram will first be processed by a batch normalization B. Then, the model learns the features from four convolutional layers $C(n_{c},k_{s})$. Here $n_{c}$ is the number of feature maps and $k_{s}$ is the kernel size. We have used a 3×3 kernel size in the convolutional layer with a stride of 1. The stride determines how many pixels the filter shifts. The depth (number of channels) of the features in each convolutional layer is shown in Figure 8. The pooling layer is used to reduce the size of the features. We have employed max-pooling which retains only the maximum value within a pool. Afterward, the flattening layer is applied, which involves transforming the two-dimensional matrix into a column matrix (vector). This column vector is then passed to the fully connected layer. We will use a non-linear ReLU function for activation.

The CNN architecture of sub-network is abbreviated as $\Upsilon_{s1}=F_{1}\left(X\right)$: B→C(16)→P→B→C(32)→P→B→C(64)→P→B→C(128), where P is the max-pooling layer. We deploy CBAM in the second module $F_{2}$, which adaptively refines the feature map, defined as $\Upsilon_{s2}=F_{2}\left(\Upsilon_{s1}\right)$. Finally, three fully connected layers $F_{3}$ are applied to encode the output of CBAM as the feature vector X. A person can be identified by calculating the pairwise distance with the template in the database.

## 4. Evaluation

In this section, we begin by outlining our experimental equipment, setup and system workflow. Subsequently, we present our experimental results and comparison with other algorithms. Finally, we conduct an assessment of each component of MeshID.

### 4.1. Experiment Setup

In principle, our approach is fundamentally applicable to a wide range of RF-based devices, e.g., WiFi, Universal Software Radio Peripheral (USRP), Bluetooth and RFID. To flexibly design carrier signals and baseband waveforms, we utilize USRP devices (Ettus Research, Austin, United States) for system implementation. Specifically, we have two USRP N210 devices (RX1 and RX2), each equipped with an omnidirectional receiver antenna. At the same time, we have two NI USRP 2953R devices with two omnidirectional antenna transmitter pairs. The transmitter sends a simple sinusoidal waveform at a fixed frequency. The transmitters are connected to the PXI Chassis(Ettus Research, Austin, United States). All of the devices are synchronized to the CDA-2990 Clock Distribution Device(Ettus Research, Austin, United States).

To capture two-dimensional CSI variance, we set the two transmitter pairs orthogonally. When performing gestures, the drawing area typically aligns parallel to the user’s orientation. Therefore, the optimal orientation is that placing two transmitter pairs and two receivers orthogonally in front of the user so that the finger could cross more double-source interference fringes. Utilizing only one transmitter pair would render the system sensitive primarily to motions perpendicular to the transmitter pair.

**Figure 9:**
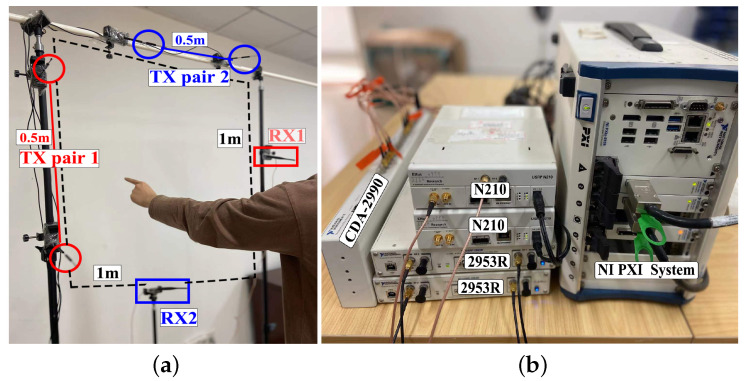
Experimental setup. (**a**) Setting. (**b**) Devices.

Specifically, the transmitter pair 1 ($TX_{1}$) operates at 5.76 GHz is placed horizontally positioned with a 50 cm apart, while the transmitter pair 2 (TX2) operates at 5.72 GHz is placed vertically positioned, also with a 50 cm apart, as shown in Figure 9a. The optimal node distance of a transmitter pair is 50 cm, which will be discussed in later Section 4.2. The distances between TX1 and RX1, TX2 and RX2 are both 1 m. Since the frequency of the TX1 pair and TX2 pair have a slight difference, the transmitted signals only interfere with each other inside each transmitter pair. The devices are shown in Figure 9b. Both receivers and transmitters are deployed on a customized shelve with an orthogonal setting. Our algorithms are performed in a DELL server with an i7-6850K 3.6 GHz Processor and 64 G RAM. The operation system of the server is Windows 10 with 64-bit.

**Figure 10:**
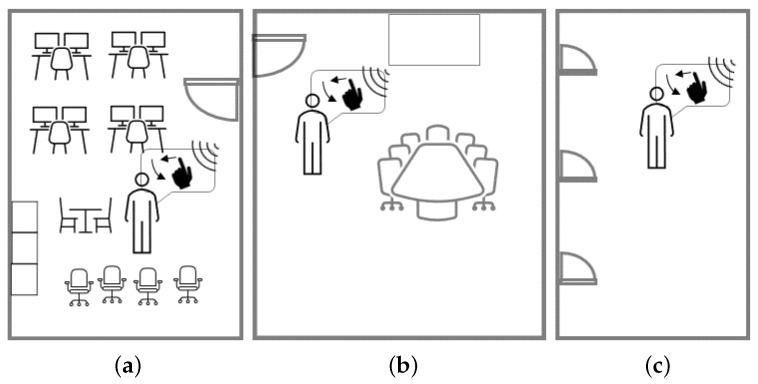
Three indoor environments. (**a**) Office. (**b**) Meeting room. (**c**) Hallway.

We thoroughly evaluate our prototype across three different indoor environments: a standard office (3.4 m × 3.8 m), a meeting room (5 m × 7 m), and a hallway (2.8 m × 35 m), as shown in Figure 10. During data collection, ambient individuals within these environments were not required to vacate the premises.

Our experiments involve the collection of two datasets. During data collection, users are instructed to freely perform in-air gestures at their desired speed and size. The first dataset contains 2268 gesture samples (6 users × 3 scenes × 6 gestures × 21 samples). We select 6 in-air finger gestures from three categories: digits, letters, and symbols for user identification, as they are the most commonly used in passwords. Specifically, we use “3” and “6” for digits, “*d*” and “*M*” for letters, and “@” and “&” for symbols. The second dataset serves to evaluate the system. The data is collected from another 18 users. This dataset comprises a total of 3600 gestures samples (18 users × 10 gestures × 20 samples). Users are instructed to perform 10 Arabic numerals “0–9” to further analyze user-to-user feature variations. Digits are a basic and familiar form of characters for most people. They are universally understood and accepted across different languages and cultures, making them accessible to a wide range of users without language barriers. In total, our dataset comprises 24 users, including 18 males and 6 females with heights ranging from 155 cm to 185 cm and weights ranging from 45 kg to 80 kg. Standard cross-validation techniques are employed in our evaluation process.

### 4.2. Impact of Node Distance between Transmitters

**Figure 11:**
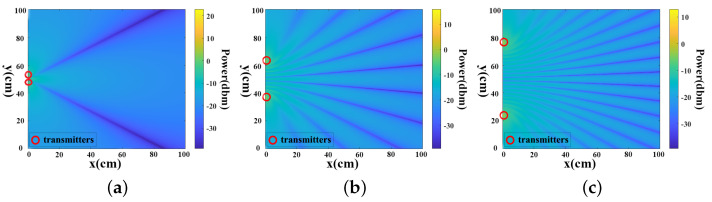
Double−source interference pattern at three different distance of a TX pair. (**a**) Distance of a TX pair is λ. (**b**) Distance of a TX pair is 5×λ. (**c**) Distance of a TX pair is 10×λ.

In MeshID, the resolution of the signal is determined by the density of the mesh, which in turn relies on the node distance between each transmitter pair. This subsection explores the impact of such node distance on the mesh pattern. Since the interference setting mandates the node distance to be a multiple of the wavelength (approximately 5 cm in our setup), within the 100 cm × 100 cm area, we varied the node distance from 1×λ to 10×λ, incremented by 5λ. The theoretical interference patterns are illustrated in Figure 11a–c. The findings indicate a direct proportional relationship between the node distance of each transmission pair and its density when the radio frequency remains constant. Opting for a denser mesh pattern necessitates a larger node distance, and vice versa.

We employ the first dateset for the subsequent two subsections, where six users are tasked with performing six gestures across three categories. To be specific, “3” and “6” for digits, “*d*” and “*M*” for digits, and “@” and “&” for symbols. Performance evaluation for gesture recognition and user identification is conducted using the standard cross-validation method. Few samples (4 in our model) of the one user are used for retraining and the rest of the samples from this users are used for testing. The data from another five users are only used for pretraining).

**Figure 12:**
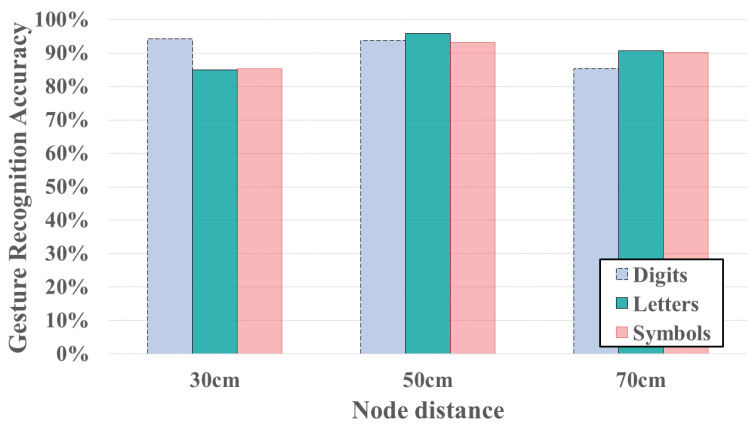
Gesture recognition accuracy under different distance of a transmitter pair.

**Figure 13:**
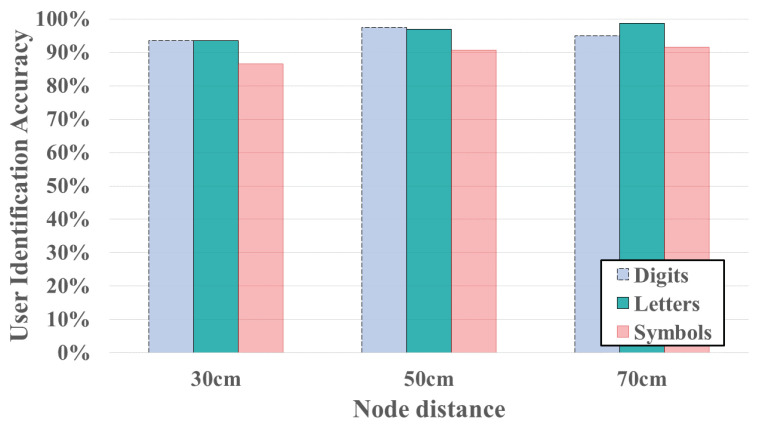
User identification accuracy under different distance of a transmitter pair.

The experiments results for three different transmitter node distances (e.g., 30 cm, 50 cm and 70 cm) are shown in Figure 12 and Figure 13. The results demonstrate that using 5 cm node distance setting achieve the highest average accuracy for both gesture recognition accuracy and user identification. In Figure 12, with the node distance set to 50 cm, the gesture recognition accuracy of three types of symbols are 93.75%, 95.83% and 93.23%, respectively. The average accuracy of gesture recognition across the three different transmitter node distances is 88.19%, 94.27% and 88.72%, respectively. Figure 13 illustrates the impact of employing three different node distances on user identification. The average accuracy for user identification in different settings is 91.12%, 95% and 95.13%, respectively. The system may fail in gesture recognition, but it still possible to identify user successfully. This is attributed to the fact that although the FCR extraction is trained based on the result of gesture recognition, but we transformed CSI time series to several components, only few top components (the first component in our FCR design) is removed. Consequently, user-specified features for user identification may still be retained. Hence, the user identification could has better performance than gesture recognition.

Additionally, the results indicate minor differences between different node distances in MeshID. Hence, the antennas placement in MeshID is flexible to accommodate various real-world scenarios with practicability. Considering our scenario is to identify the users through finger gestures, we need to make sure that the mesh cell size is able to match the finger width (approximately 2–3 cm) of an average person. Therefore, we default to setting node distance as 10×λ (λ=5 cm) by default in our subsequent experiments.

### 4.3. Performance of MeshID

Table 1 presents the results of gesture recognition and user identification across three different environments, spanning from a compact office space to an expansive hallway. These assessments utilize the first dataset and adhere to the standard cross-validation procedure. Results reveal that the gesture recognition accuracy of MeshID achieves 94.27% in an office, 94.1% in a meeting room and 93.4% in a hallway. Notably, since the differences of the results across these environments are minor, MeshID is able to achieve a fair robustness in adapting to diverse indoor settings. MeshID attains identification accuracy of 94.3% in an office, 95.03% in a meeting room and 97.1% in a hallway, respectively. Notably, the highest identification accuracy is recorded in the hallway environment, attributed to the minimal presence of multipath effects. On average, MeshID system achieves an identification accuracy of 95.48%. According to the results, the identification accuracy on average of three gesture categories is 95.25% for digits, 95.52% for letters, and 95.67% for symbols. It can be observed that all types of gestures perform well in three indoor environments. Delving into specific gestures, the average accuracy of gesture “3” is 93.3%, gesture “6” is 97.2%, gesture “*d*” is 97.5%, gesture “*M*” is 93.5%, gesture “@” is 99.1% and gesture “&” is 92.2%. Digits are universally understood and accepted across different languages and cultures, making them accessible to a wide range of users without language barriers. Therefore, to make the evaluation more general, we choose digits as the identification gestures for the following experiments.

### 4.4. Performance of User Identification

**Figure 14:**
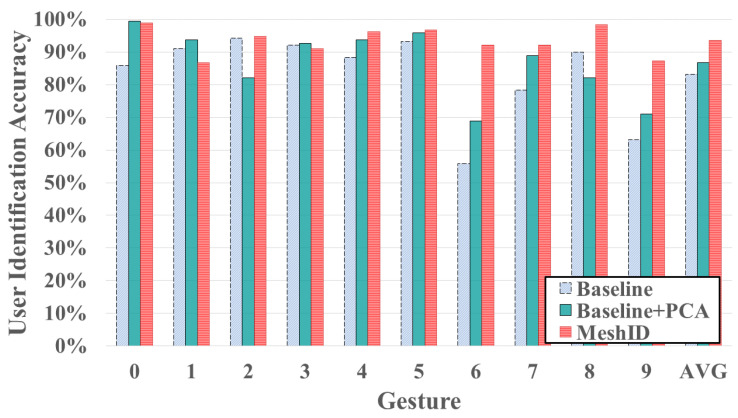
Comparison of key components.

To further investigate the performance of our user identification system, we employ a larger second dataset for subsequent evaluations. 18 users are asked to perform the same finger gestures 20 times. Data from the remaining 6 users are only used for intrude detection in later. Other 12 users are evaluated using the standard cross-validation method. Without loss of generality, we test digital numbers (“0” to “9”) 20 times for each user. Consistency was maintained in the stroke order for each digit, with users instructed to write the digits in a clockwise manner (e.g., writing digit “0” clockwise). The user identification results are shown in Figure 14. The average identification accuracy across the 10 gestures stands at 93.19%. With the exception of digit “1”, the accuracy of all other digits exceeds 84%. Specifically, four digits demonstrate outstanding accuracy: 98.9% for digit “0”, 96.3% for digit “4”, 96.8% for digit “5”, and 98.4% for digit “8”. Conversely, the identification accuracy for finger gesture "1" and "9" is comparatively lower than others. This discrepancy could be attributed to the simplicity of the strokes for “1” and “9”, where different users might not exhibit significant variations in their finger gestures. Conversely, for the remaining finger gestures, we observe a notably high identification accuracy.

To further evaluate the security level of the system, we conducted an intrusion detection scenario where we enlist 6 unseen users to act as spoofers. These 6 spoofers are replicate the gestures of target users in an attempt to bypass the user identification process. We employ the true negative value to measures the probability that MeshID correctly identifies an unauthorized user. The results, presented in Table 2, reveal a detection accuracy exceeding 80% for all 6 spoofers, with four of the six users achieving approximately 90%. While our primary focus lies on user identification rather than binary classification, the framework of MeshID could still achieves an overall detection accuracy of 91.7% with no prior information of the testing environments.

### 4.5. Impact of Signal Interference

As mentioned in Section 3, we have learned in theory why signal interference can improve the sensing sensitivity. To further understand how the signal interference affects the performance, we conducted a comparative analysis of experimental results using spectrograms based on our double-source interference setting and the traditional single-source setup. We transform the CSI signals obtained from both settings to Continuous Wavelet Transform (CWT) spectrograms. Since the VDPs serve as a unique feature based on interference patterns, we opted for the more commonly used CWT for the comparison.

In single-source experiments, only one ominidirectional antenna is connected to a transmitter, resulting in no signal interference within the finger movement area, in contrast to the double-source setup. In double-source interference scenario, two ominidirectional antennas, referred to as an antenna pair, are connected to a transmitter. Both antennas operate under the same transmission settings, including frequency. Data from 10 users of second dataset are used in this experiment. The other setting remains consistent with those previously introduced.

**Figure 15:**
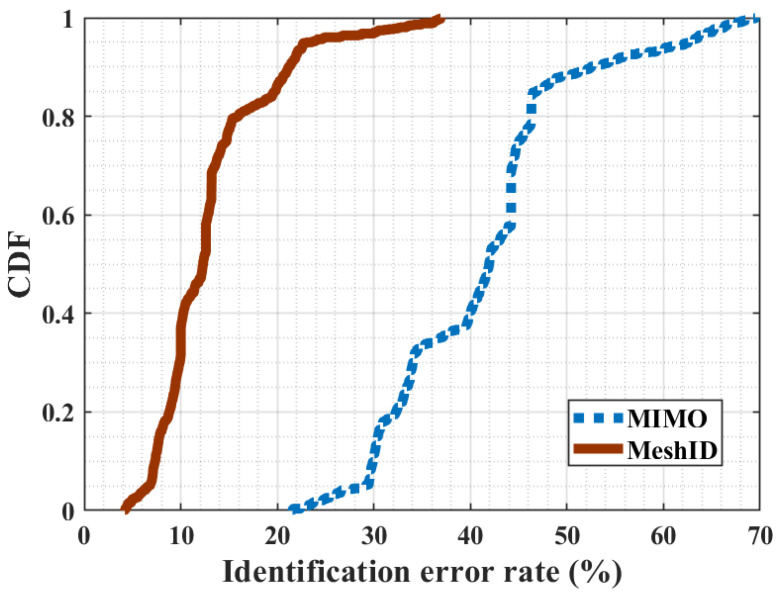
CDFs of MIMO and MeshID.

Figure 15 illustrates the Cumulative Distribution Function (CDF) of the identification error rate with interference and without interference. We can see that the average accuracy of double-source interference setting significantly surpasses that of the single-source setup. Specifically, the inclusion of interference in the signal enhances the system’s identification accuracy by 28.3% compared to scenarios without interference. Therefore, double-source interference setup creates a fine-grained signal mesh within the designated area, leading to high user identification accuracy.

### 4.6. Impact of Ambient People Moving

**Figure 16:**
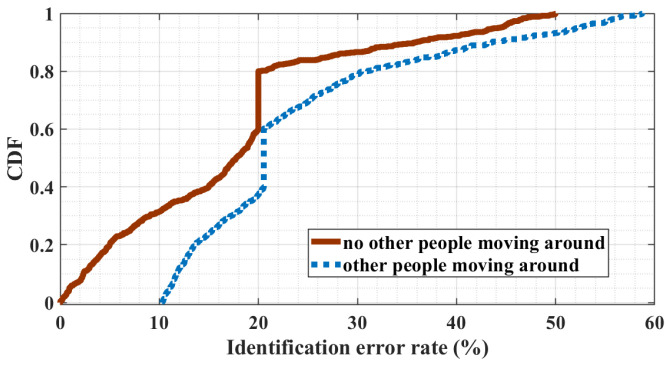
User identification error rate of user performing one gesture when ambient people walk around.

**Figure 17:**
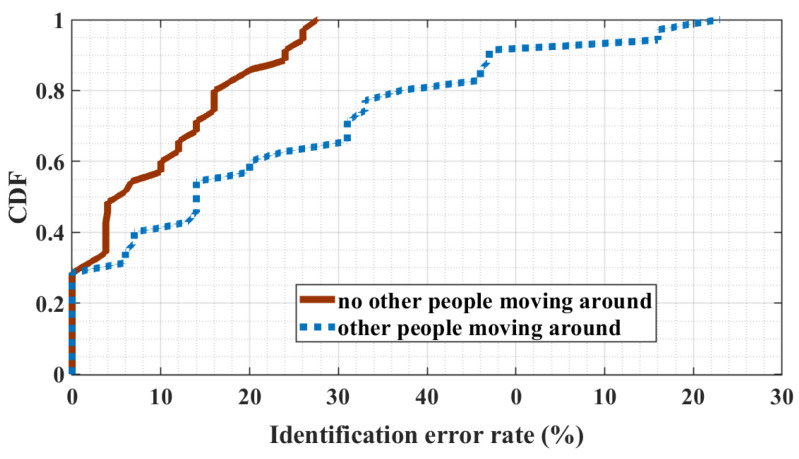
User identification error rate of user performing two sequential gestures when ambient people walk around.

In a typical public room, it is a common condition that other people may dynamically move around when the user is performing finger gestures. Since the wireless signal is sensitive to ambient environment changes, ambient people moving may easily affect the CSI variances, which may have a side effect on user identification. We study the performance of MeshID under the impact of ambient people moving. Figure 16 showcases identification results when a single gesture is performed while an individual moves within a 3-m range. Similarly, Figure 17 depicts results when two consecutive gestures are executed amidst ambient movement. The presence of ambient movement causes a slight decrease in identification error rates, which remain within acceptable bounds for most scenarios. Furthermore, increasing the number of finger gestures performed by the user enhances identification accuracy. This result demonstrates that MeshID is performed as robust to the impact of ambient people moving. Theoretically, the proposed interference wave, comprising a superposition of two waveforms with same frequency, results in stronger CSI variances, facilitating more resilient feature extraction for user identification compared to traditional single-wave setups. Consequently, MeshID effectively mitigates the adverse effects of ambient movement, enhancing overall robustness.

### 4.7. Performance of FCR Extraction and CBAM

FCR extraction and the CBAM Module are two key components of MeshID. The former removes the non-related features from the input feature space, while the latter extracts features from both channel and spatial axes and focuses on the places with more important information. In order to investigate how these two components affect the performance, we compare our algorithm with a traditional CNN and test the impact of each component.

As shown in Figure 14, employing only the basic CNN yields an average accuracy of 83.2%. Introducing the PCA reverse extraction algorithm enhances the average accuracy to 86.8%, surpassing the basic CNN by 3.6%. Furthermore, integrating both the PCA reverse extraction algorithm and the CBAM Module elevates the average accuracy to 93.5%, marking a 6.7% improvement. Therefore, both components significantly contribute to enhancing user identification accuracy.

### 4.8. Comparison with Baseline Approaches

MeshID could be a more robust and flexible extended authentication component in existing recognition systems. We evaluated our system on user identification by comparing it with two alternative state-of-the-art approaches, FingerPass [38] and FreeAuth [18]. Both of them are leveraging wireless information for user authentication. Specifically, FingerPass utilizes segmented CSI time series as learning features and adopts the LSTM-based DNN model. FreeAuth proposes a CNN-based method for extracting CSI features. FreeAuth applies a Recurrent Neural Network (RNN) model to extract users’ gesture characteristics and maximize the individual uniqueness characterized by a Gaussian Mixture Model (GMM). To control the variable, the training and evaluation process for the baselines follow the same rules of MeshID (e.g., the number of training epoch). We utilize the same dataset, which is our first dataset, for training and evaluation purposes. The comparison results of user identification are summarized in Table 3. Both MeshID and FreeAuth exhibit superior identification results, achieving over 90% accuracy, compared to FingerPass when tested with seven users.

To ascertain the relationship between system performance and the number of users, we evaluated the three systems with varying numbers of users, ranging from 7 to 12. FingerPass and FreeAuth experience a significant degradation in identification accuracy as the number of users increases. However, our system maintains an average identification accuracy of over 90% for up to 12 users. MeshID achieves better overall performance than the other two approaches, specifically, outperforming FingerPass by about 6% and FreeAuth by nearly 30% with up to 12 users. We noticed that emphasizing user-specified features can mitigate the side effect of the same gesture, and improve the robustness of the system with a larger user number.

It satisfies the demands of most families and some small groups. The performance of MeshID keeps consistency as the number of users increases from 7 to 12. In reality, the challenge of user heterogeneity increases significantly with a large number of users (e.g., thousands of users). However, collecting and labeling finger gestures from such a massive user pool for evaluation purposes poses considerable‘difficulties.

## 5. Discussion

**Figure 18:**
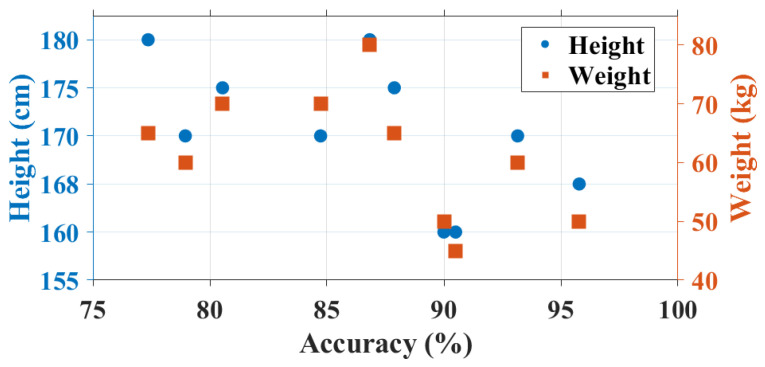
Statistics of users.

Impact of user height. Different user height may impact the identification accuracy. To investigate the relationship between identification accuracy and user height, we analyzed the height distribution of users. As the statistical result shows in Figure 18, shorter users tend to achieve higher identification accuracy. The height distribution, ranging from 155 cm to 185 cm, is represented on the left axis. In our experiments, we fixed antenna pairs at relatively low positions to accommodate users of different heights. Consequently, taller users may experience more reflected signals from lower parts of their body, such as the chest. Despite this challenge, MeshID maintains a high capability of identification, as gesture pattern retain their uniqueness with interference settings. However, it’s worth noting that antenna height adjustments can be made to suit different scenarios.

Impact of user weight. The weight of the users varies from 45 kg to 80 kg. The right axis in Figure 18 represents the weight of the user. The figure shows that the identification decrease is not caused primarily by user weight. Although some of the statistical values show that identification accuracy is better when user has lower weight. That is because user who is shorter usually has lower weight. To delve deeper into this relationship, we conducted a focused analysis on data from users within the same height range. Surprisingly, our findings reveal no discernible pattern in the distribution of identification accuracy, indicating that weight alone does not dictate accuracy levels.

Impact of motion speed. In our experiments, users are free to perform gestures de-pending on their habits. Different motion speeds may result in different sample lengths.However, RF devices usually have a related high sampling rate (e.g., more than 250 Hz), hence the sampling interval is less than 0.004 s. It is short enough to capture the normal motion finger. The sampling rate of USRP is much higher; it could be up to 200 MHz. In our setup, the signal sampling rate is 651 Hz, It is adequate for different motion speed samples. There-fore, the motion speed is not the significant factor that affects the identification accuracy.

Impact of different environments. The multipath effect is a phenomenon prevalent in radio frequency applications where signals travel multiple paths from transmitter, encountering reflections, diffraction, and scattering off objects and surfaces in the environment. Consequently, the receiver detects various versions of the transmitted signal, each arriving at slightly different times and exhibiting diverse amplitudes and phases. In principle, RF systems in outdoor environments usually perform better than indoor. Since in indoor environments, with numerous reflective surfaces and obstacles, the multipath effect becomes particularly pronounced, leading to signal degradation and complicating signal processing. We conducts MeshID in three environments: an office, a meeting room and a hallway. According to the identification results, which are 94.3% in the office, 95.03% in the meeting room and 97.1% in the hallway. These outcomes suggest that reduced multipath effects likely contribute to the system’s enhanced performance.

Latency. It takes about 1 s to 30 s to perform a gesture in our evaluation. This timeframe is sufficient to satisfy most users, including those with physical limitations who can only draw gestures slowly. We also implement MeshID on a desktop with 12th Gen Intel(R) Core(TM) i3-12100F 3.30 GHz. The time consumption of MeshID mainly comes from the noise removal module and the data transformation module. It demands, on average, 1.743 s to identify the user when eight users are in the database after segmenting the gesture. We believe it is adequate for the majority of identification application scenarios for users.

From the results, it is convincing that the proposed mesh and one-shot DL approach can address the challenge of user heterogeneity, enabling MeshID with high robustness.

## 6. Related Work

### 6.1. Behavioral Identification

Behavioral identification as a subset of biometric authentication has been well pro-posed using a range of sensing technologies. Vision technology [39,40] can identify different users through physical activity characteristics captured from image frames using high-resolution cameras, but it may easily fail in the conditions of luminous changes and obstacles placed in line-of-sight (LoS) [4], in particular raising severe user privacy concerns. Bioelectrical technology [41,42,43,44] can utilize bioelectrical sensors, e.g., electrocardiogram (ECG), electromyogram (EMG) and electroencephalogram (EEG), to precisely extract unique biomedical information through body’s electrical activities. Ashraf et al. [45] propose a fusion system that uses biometric features of the iris and foot. It achieves a very high accuracy of 98%. Ref. [46] utilizes the phase transform method and Fourier decomposition method to identify individual ECG features. Since these sensors are required to be attached carefully to the user’s body, such wearable requirements may compromise user experience, leading to inconvenience in reality [47]. In contrast, since our identification system is essentially developed by RF technology, MeshID can enable user-friendly device-free identification with the advantages of being illumination-free and having fewer privacy concerns.

### 6.2. RF-Based Behavioral Identification

Existing works focus on different individual behavioral characteristics [10,15], e.g., daily activities, walking gaits, vital signs, and gestures. For gait-based identification, WiWho [48] uses commercial WiFi devices to verify a small group of users using walking gaits. WiFi ID [49] explores the relationship between the feature pattern of subcarrier-amplitude frequency (SAF) based on WiFi CSI and individual walking style, and employs a linear-kernel support vector machine (SVM) to identify users. For daily activity-based identification, E-eye [50] proposes to identify users using the WiFi CSI profiles caused by the activities across the home on a mobile device, while Au-Id [5] uses the reflected RFID signal of users’ activities for identification. Since these works usually require a long-term user activity tracking, the real-world applications still remain limited. Besides, a numbers of works propose to enable gait-based identification. Shi et al. [51] extract WiFi features of walking and stationary activities for human authentication. RFree-ID [52] identified human targets based on walking gait by using a phase matrix from tag array. Due to the large range of gait motion, these works may be strongly vulnerable to the impact of ambient environments. Similarly, some works demonstrate vital sign based identification to estimate users’ heartbeat rate [16,53,54] and respiratory biometrics [17,55] from RF signals, but the identification may practically fail as the motion of vital signs remains brittle to body movement artifacts and ambient noises. Since performing gestures is user-friendly in a small motion area, gestures-based identification is promising to have much less impact on ambient noises. WiHF [19] is proposed to recognize gestures using WiFi signals for enabling user identification, but the proposed gestures still remain as a large range of arm motions, resulting in much less user-friendliness compared to using in-air finger gestures. FingerPass [38] proposes to identify users through finger gestures with fair accuracy, but the identification performance may still be subject to the issue of low RF signal resolution due to using a traditional RF setup. Unlike these two works, MeshID leverages on the effects of orthogonal signal interference and an attention-based siamese network to fundamentally improve the signal resolution and model retraining, achieving high identification accuracy and robustness.

## 7. Conclusions and Future Work

This paper presents a novel RF-based user identification scheme that leverages on the proposed mesh and few-shot deep learning approaches to enable highly accurate user identification through finger gestures. MeshID can essentially promote the sensing sensitivity on RF signal to extract sufficient individual biometrics from the movements of finger gestures, accurately identifying different users even in a complex environment. Also, MeshID is able to efficiently retrain the model to ensure high robustness, adapting to an unseen user with little data. In practice, MeshID as an appealing add-on can be easily integrated into existing RF-based gesture recognition systems at low cost. To further investigate the robustness of MeshID, we plan to test our approaches on existing large finger gesture datasets, and develop MeshID for efficient integration with existing RF-based gesture recognition systems. Other methods [56] such as SVM, Logistic Regression and Random Forest will be considered as the improving module in our system. Also, we plan to further evaluate the robustness of our prototype in more real-world scenarios in our future work.

## Figures and Tables

**Table 1:** MeshID performance in different environments.

Recognition	Gesture	Office	Meeting Room	Hallway
Gesture Recognition	Digits	93.8%	95.8%	93.2%
	Letters	96.9%	92.2%	93.2%
	Symbols	93.2%	94.8%	92.2%
User Identification	Digits	92.9%	97.4%	95.5%
	Letters	90.9%	97%	98.8%
	Symbols	99.2%	90.8%	97.1%

**Table 2:** Detection accuracy of spoofer.

Spoofer	s1	s2	s3	s4	s5	s6	Avg
Detection accuracy	95%	95%	85%	100%	80%	95%	91.7%

**Table 3:** Method Comparison with Different User Number.

	System	FingerPass	FreeAuth	MeshID
User Number				
7 users		83.6%	93.93%	97.4%
8 users		78%	87.24%	96.5%
9 users		76.8%	70.76%	95.3%
10 users		71.1%	68.69%	94.7%
11 users		62.6%	67.0%	94%
12 users		60.9%	66.3%	93.4%

## Data Availability

Data will be made available upon request.
